# Physiological Psychology

**Published:** 1856-07-01

**Authors:** Robert Dunn


### Art VI.—PHYSIOLOGICAL PSYCHOLOGY.

### No. II.

BY ROBERT DUNN, F.R.C.S. ENG.

(*Continued from page* 240.)

The scientific procedure of psychology, according to Fichte, essentially consists in separately considering the *intelligence*, the *feelings*, and the *will*, and in carefully observing and studying their parallelism in the different stages of mental development. We have considered the unity of the mind in *self-consciousness*, —its earliest, and consequently lowest, phase of development—*in sensori-motor, consensual, and instinctive feelings and actions;* where the *intelligence* is purely sensational, the *feelings* simply those of pleasure and pain, and the *impulses to action* inherent and instinctive.

We have now to consider it in the *perceptive consciousness*, the next stage of our psychological progress, in *ideation*, *emotion*, and *volition;* and here, too, there exists a perfect unity at the root, from these being so closely interwoven with each other. For without ideation there can be no determinate or voluntary action, and without the will no act of intelligence; while alike with both and with either, emotional sensibility is indissolubly connected.

The genesis of the will is in the perceptive consciousness, and it proceeds, *pari passu*, with the development of the intellectual faculties, until they reach their dominant development—the highest reason and the freest will;—and then it is that an act of the will embodying the whole man emphatically implies, at the same time, *intelligence*, *emotion*, *impulse*. But when the perceptive faculty is in abeyance, the will is in abeyance, and memory is abolished. Of this we had a striking illustration in the young woman's case, to whom I have before alluded. In her the mental faculties were quite suspended, and all the avenues to the sensational consciousness were closed, with the exception of sight and touch, for she could neither hear nor speak, smell nor taste. Her mind was in a state of isolation, and even through sight and touch no *ideas* were aroused, for the perceptive faculty was in abeyance, but the will was in abeyance also, and memory she had none. "She had no notion that she was at home, nor the least knowledge of anything about her. She did not even know her own mother, who attended upon her with the most unwearied attention and kindness. Wherever she was placed there she remained throughout the whole day, making not the slightest *voluntary* effort of any kind, manifesting no uneasiness for anything to eat or to drink, and taking no heed whatever of what was going on around her." In fine, while the perceptive faculty was benumbed and paralysed, *ideation*, *memory*, and *volition* were alike abolished.

*Perceptive Consciousness.* Sensory impressions, the intuitions of the special senses, whether sights, sounds, smells, tastes, or feelings, internal or external, in order that they may reach the perceptive consciousness, and so become *idealized* and *registered*, require to be transmitted from their respective sensory ganglia to the great hemispherical ganglia, or cerebrum, for it is there that *ideation* is effected, and *memory* resides. But if, indeed, the perceptive faculty should become suspended, then "all the enjoyments of the feast, all the fragrance of the flowers, and the whole of the associations which they embody, vanish as with a single and magic stroke."* And, as in this young woman's case, the most nauseous medicines would be taken quite as readily as the most delicious viands. Such, too, is the fate of all our associations in connexion with the higher and more objective of the senses, with *hearing*, *feeling*, *sight*. For the whole world of tone,—the grandest harmony, the softest melody, the living voices of nature, —exist not when the percipient power is in abeyance; nor without its agency can our tactile sensibility impart to us any knowledge of the bodily substances by which we are impressed, or identify the impressions with the forms of the external objects that produced them. And as for light—to what do the intuitions of light and colour amount without the perceptive faculty, and what the pictured image on the retina without the perceptive organ beyond it? To the eye, without the perceptive faculty behind it, "the universe would be all dark and dreary, not a tint or a hue there, not a smile on the face of nature, nor a shade of beauty on the summer's landscape."† And thus it is that perception is the portal to intellectual action; for while in *sensation*, the conscious mind feels intuitively the physical impulse of the outward object as it affects the consciousness through the sensorium, in perception the nervous impression is carried a stage farther, and by virtue of the harmony which exists between the percipient mind and the external world or nature, the sensory impression is intuitively translated into the form of intelligence, and becomes an intellectual phenomenon; in other words, it is *perceived* and *idealized*. The process in both cases is equally and alike intuitive. For when we look at an external object, we can no more avoid the perception that it is a something distinct and apart from ourselves, and of having forced upon our minds intuitive ideas as to its size, shape, colour, &c., than we can reject the sensations of touch, as to its hardness or softness, or those of taste as to its sweetness or bitterness, or of smell, as to its fragrance or offensiveness; in each and in all, the

* Morell's Psychology. † Ibid.

process is alike intuitive. But these two states, nevertheless, of consciousness,—*sensation* and *perception*,—though both intuitive and so closely allied, are not to be confounded, for they are *distinct*, and the mechanism (so to speak) of their action is *different*. The one is a single, and the other a complex act. In *sensation* it is direct and single, for the impressions made on the sensory ganglia go direct to the sensational consciousness; but *perception* is a step in advance in our psychological progress, *above* sensation, and in it a double ganglionic action is involved. For the sensory impressions to become *perceived*, that is, *idealized* and remembered, they require to be transmitted from the sensorium to the cerebrum, "the sole receptacle," in the language of Cuvier, "where the various sensations may be, as it were, consummated, and become *perceived* by the animal, and where all sensations take a distinct form, and leave lasting traces of their impressions, serving as a seat to memory, a property by means of which the animal is furnished with materials for its judgments."*

In illustration of this view, Dr. Noble† has well observed:—

> "An anatomical distinction between the region of *thought* and that of *sensibility* can very fairly be established; and a certain aptitude, moreover, can be recognised in the encephalic structure for conveying the impressions of the senses upwards to the hemispherical ganglia. White matter intervenes between the vesicular neurine of the sensory ganglia and that of the cerebral convolutions; the conscious impressions received by the former may be regarded as ascending along the white fibres, and, on the gray summit being attained, developing changes in its condition which minister to intelligence. *Ideas arise.* If we reflect upon the processes that go on within our own minds, there is no difficulty in distinguishing between a *sensation* and an *idea*, or in marking the sequential origin of the latter. How often do we find that, when the full consciousness of sensation is obtained, the idea suggested by it does not follow until some seconds, or even minutes afterwards. For example, you hear the utterance of certain words as sounds; their signification does not strike you; no effort of attention is made, yet suddenly the sense breaks upon your intelligence. The correlated physiological phenomena may be thus stated. The auditory ganglia take up the sentient impression at once; its passage onwards to the seat of thought is delayed; presently, however, its natural course is freed, as if from some hindrance, and it attains the hemispherical ganglia, forming or awakening ideas in the mind."‡

* Cuvier, *Rapport sur le Mémoire de Flourens sur le système nerveux*, quoted by Dr. Todd. *Vide* "Cyclopædia of Anatomy and Physiology, and Functions of the Nervous System."

† *Vide* Dr. Noble's Lectures "On the Co-relation of Psychology and Physiology," page 27.

‡ The *modus operandi* of Anæsthetic agents, in relation to their action upon the different nervous centres of the encephalon, is highly interesting and instructive. It brings strong confirmation to the important facts, that sensation and perception are *distinct states* of consciousness, that they have their seat in different nervous

Before entering, however, upon the consideration of the phenomena of the perceptive consciousness, and of the local habitation of its organs in the cerebrum, I think we shall proceed with decided advantage, in reference to the physiological bearings of the subject, seeing that throughout the entire vertebrate subkingdom, the type of the brain is the same,—if we first pass in review the whole of the ganglia of the encephalon, and endeavour to specialize their functions, beginning with the lowest of the centres, and that the sensational consciousness may be suspended, while the perceptive remains intact. As bearing on these points, I brought the subject of "*the inhalation of chloroform, its anæsthetic effects, and practical uses,*" under the notice of the Royal Medical and Chirurgical Society, in a paper, which was read and discussed, April 22, 1851, and afterwards published in the *Medical Gazette* of the same year. In illustration, I may here cite the following paragraphs:—

"There can be no doubt that the anæsthetic effects of the inhalation of the vapour of chloroform are due to its entering the circulation, and to its being carried by the blood to the vesicular matter of the sensory ganglia, and to the cells, or cell nuclei, at the peripheral extremities of the afferent nerves. And while it is reasonable to infer that, in thus circulating with the blood through the encephalon, its presence, like that of any similar morbific agent, must more or less affect all the sensory feelings and psychical manifestations, it is nevertheless abundantly manifest that a kind of elective affinity exists, by virtue of which the vesicular matter of one centre of action becomes affected before that of another; for, during the slow and gradual inhalation of the vapour, the function of sensation is suspended before that of intellectual action,—the *consciousness of feeling* is obliterated, and consequently immunity from pain secured, before *intellectual consciousness* is totally abolished. M. Flourens was, I believe, the first to point out the tendency of certain morbific agents to act primarily and specially on one nervous centre in preference to that of another, by virtue of some special elective affinity between such agents and certain ganglia of the encephalon.

"From the records of personal experience, and from a careful consideration of the phenomena observed in others, we may trace the following order and sequence in the effects of the inhalation of the vapour of chloroform, properly diluted, upon different nervous centres.

"Thus, the first few inhalations are attended with *feelings* which indicate disturbance in the action of the sensory ganglia, as 'singing in the ears, a sense of numbness, and tingling of the surface of the body,' &c., but which are soon succeeded by a *transient stage* of more general excitement; of delirium in the hemispherical ganglia, for instance,—as singing and incoherent talking, and of excited emotional impulses, and consensual movements in the *sensory ganglia,*—as laughter and uncontrollable motorial actions; this is speedily followed by suspension of the function of sensation, *the consciousness of feeling,* while as yet some degree of *intellectual activity* remains. Sensorial impressions *from without* are no longer transmitted from the sensory ganglia to the cerebrum; but this 'suspension of ordinary sensational impressions, as in sleep, with persistent intellectual activity, is the *typical* characteristic of dreaming;' and dreams often occur. The commissural fibres, between the cerebrum and these ganglia, *Reil's nerves of the internal senses,* being still in action, they transmit downwards the *residual intellectual activity* from the cerebrum to the sensory ganglia, and frequently give rise to manifestations, which impress the mind of common observers with the belief of pain and suffering being felt under the knife of the surgeon, while in reality there are none.

"The function of the cerebrum as the centre of intellectual action is next suspended; a state of coma is induced, *a complete abolition of consciousness, reducing life to a series of automatic movements.* After this the medulla oblongata and true spinal centres become involved, reflex action is stopped, and breathing by the ribs suspended. The ganglionic system is the last to be implicated; but, with the arrest of the peristaltic action of the heart, life ceases."

vertebrate series, and thus "making use of the lower animals, as so many experiments ready prepared to our hands by nature." In man, indeed, the cerebrum is so enormously developed, that it completely overlaps and crowns the other encephalic ganglia, whilst in the lowest of the series its representative is reduced to a mere lamina or crust. Now, proceeding in this way, if we advert to the brain of the fish, the lowest in the series of the vertebrate sub-kingdom, and where there exists the least complexity of structure, what do we find? And of what are the ganglionic bodies which we do find, the *homologues* in the human encephalon? We find, in the brain of the fish "a series of at least four distinct ganglionic masses, arranged in a line continuous with the spinal cord, three of them in pairs, and the last or hindmost single." Respecting these, a rigid scrutiny and a strictly philosophical induction has fully established the following important deductions—viz., that the first of these masses—the most anterior on either side of the median line—is the olfactory ganglia, the centre in which the olfactory nerve terminates, and in connexion with the anterior extremity of the medulla oblongata.

The second pair are the sole representatives of the cerebral hemispheres, but not in their totality. The exterior covering only indicates the presence of the anterior lobes, for the interior mass, from its connexions and aspects, is the homologue of the corpus striatum.

The third are the optic lobes, the ganglionic centres of the optic nerves, which contain the homologues of the corpora quadrigemina and thalami optici of the higher vertebrata. The fourth and single mass, placed over the divergent space of the fibrous strands of the medulla oblongata forming the fourth ventricle, is the cerebellum, sometimes having rudimental lateral appendages. Now the fact is indisputable, that in the early human embryo, as in the brain of the fish, the encephalon consists of a like series of distinct ganglionic bodies, amongst which the representatives of the cerebral hemispheres are usually the smallest. We have—

1st. The olfactory ganglia.

2nd. The corpora striata, covered by their laminæ, which are the rudiments of the cerebral hemispheres.

3rd. The thalami optici, inclosing the third ventricle.

4th. The corpora quadrigemina, and

5th. The cerebellum.

It has been truly observed by Dr. Carpenter,—

"There is no more general fact in the whole range of comparative anatomy, than that the encephalon of the vertebrata is composed of *these elements*, at the commencement of its development, and that the whole history of the evolution of the human brain indicates its precise accordance with this general type of structure."

But a searching scrutiny unfolds and demonstrates that the same distinctness exists in the nervous centres or ganglionic bodies, in the *adult brain*, as in the *embryonic;* and that the greater complexity of structure in the former is entirely due to the size and development of the cerebral hemispheres, and to their extensive commissural connexions with the other encephalic ganglia, and of those ganglia with each other.

But, adverting again to the brain of the fish and to that of the human embryo, we see that the first of the ganglionic bodies—the olfactory—are in direct fibrous connexion with the medulla oblongata; and, in the adult brain of the human subject, as I have already observed, the peduncles are also connected with the thalami optici, and with the primitive and fundamental convolutions of the cerebrum which surround the Fissura Sylvii.

In the second pair of ganglia, alike in the brain of the fish and in that of the human embryo, we find the corpora striata in close connexion with the rudimental cerebral hemispheres, forming, in fact, rounded masses with them. Now, this fusion, as it were, or rather, bending up together in the same mass, of the *motor* with *the perceptive centre*, is interesting and instructive, inasmuch as it not only indicates the closeness of their union, but presents to us, in the case of the fish, the *earliest instance* to which we can point of clear and distinct evidence of the exercise of *perception*, *memory*, and *volitional movements*, as opposed to *mere consensual actions.* But waiving, for the present, the further consideration of the *perceptive faculty* and its *cerebral organ*, I would observe, that I hold it to be indisputably established—and my own pathological researches have confirmed me in the opinion—that *the corpora striata are the motor ganglia of the encephalon.* Implanted upon the motor tracts of the crura cerebri and medulla oblongata, *in them the motor fibres terminate;* and they thus, with the vesicular matter of the locus niger and the anterior segmental ganglia of the spinal cord, constitute the motor axis of the cerebro-spinal system, and are the source of all the movements of the body, whether reflex, consensual, emotional, or voluntary. The corpora striata are not the *seat* of *volition itself*, but the encephalic motor centres, through which the mandates of the will or volitional power of the hemispheres are propagated —*the connecting links of thought with action,—of the mental with the motor forces.* Their commissural connexions with the cerebrum are so intimate and so extensive, that they are evidently placed in subserviency at every point, through the agency of innumerable radiating commissural fibres, to the volitional power of the hemispheres, in every voluntary act and effort. And thus we find, in hemiplegic patients, that the imperfect power of utterance which we so constantly meet with, is due to some structural lesion, either in these commissural fibres, or in the motor centres,—the corpora striata, through which the volitional impulses operate in speech. But the corpora striata are not solely the motor centres of volition. From their close commissural relations with the thalami optici, they are also and equally the centres and channels of respondent sensori-motor actions, and of consensual, instinctive, and emotional movements.

Dr. Todd and Mr. Bowman have clearly shown that there exists between the corpora striata and the thalami optici a relation analogous to, and as close as, that which subsists between the *anterior* and *posterior* peaks of grey matter in the cord; and as, in the case of the spinal cord, the anterior peaks, or segmental ganglia, issue motor impulses in respondence to sensations excited through the posterior peaks,—so, too, in the case of the encephalon, the corpora striata propagate motor impulses in respondence to excited internal feelings and emotions, of which the thalami are the seat, and often quite independently of volition or thought.

The spinal cord itself, though in such intimate, direct, and continuous connexion, through the medium of its cranial prolongation,—the medulla oblongata,—with the corpora striata and thalami optici, is nevertheless manifestly *a distinct and independent centre of action*, consisting of a series of segmental ganglia and nerves, structurally homologous, and functionally analogous to the jointed ganglionic cord of the articulata. The excito-motory and reflex actions of which it is the seat, are evidently subservient to the conservation of the organism, by the excitation of the respiratory movements, by the governance of the various orifices of ingress and egress, and by the maintenance of the integrity of other vital processes in which the reflex movements are concerned. And while I would here give free expression to my admiration of the genius of that able and acute investigator and discoverer, Dr. Marshall Hall, in his capacity alone of expounder of the *doctrine of reflex action*, and of its practical application in the elucidation of morbid symptoms and to therapeutics, and to my conviction of the great obligation which medical science lies under to him, I cannot plead ignorance of the fact, that many of our most eminent physiologists are opposed to his hypothesis, of the *existence of a distinct and special system of incident and reflex nerves for the production of excito-motory actions.* They maintain that muscular movements, whether reflex, emotional, or voluntary, are immediately called into action by the *same afferent nerve fibres*, and that the very *same efferent*

or *excito-motory* fibres are alike the channels for the transmission of stimuli which give rise to reflex actions in the cord—and of impressions which become sensations when transmitted to the sensorium. Nor is reflex action peculiar to the true spinal system; for it is equally an attribute of the sensori-motor, emotional, and cerebral systems.*

But, to proceed. The third pair of ganglia are the optic lobes. In the brain of the fish, the optic thalami and corpora quadrigemina are contained in one mass, forming these lobes, and presenting, in point of magnitude, a striking contrast to their rudimentary cerebral hemispheres. This fusion is interesting and instructive, and harmonizes well in fishes with the activity of their sight, and the character of their consensual movements. In the human embryo, however, the vesicles are distinct; and the thalami optici, in the adult brain, to use the words of our great physiologist, Sir Charles Bell, "forms a nucleus around which the corpus striatum bends." The thalami are the essential ganglia of the *sensory* tracts, as the corpora striata are of the motor. Implanted upon the sensory tracts of the crura cerebri and medulla oblongata, *in them the afferent fibres terminate.* They are the great centres of sensibility, for they are in direct and continuous commissural connexion with the posterior segmental ganglia of the spinal cord; and the impressions which are received by these ganglia from the sentient extremities of all the different nerves distributed upon the whole surface of the body, pass up to the thalami, and there become sensations. But they are not thus the mere centres of *common sensation;* for, as we have already seen, a continuous nervous thread ramifies throughout the entire circle of special sensation, so that the thalami are the common foci and points of union for all the sensory nerves; and this harmonizes well, as I have before observed, with the universality of the feeling, or common sensibility, which pervades the whole system, and which is associated with all the voluntary movements of the body, and with the exercise of the functions of all the other special organs of sense. In a word, the thalami optici are the *great centres of sensorial feeling,*—those points of unity around which our sensational feelings, from the earliest period, are gradually marshalled, in the development of self-consciousness,—the primary form of which essentially consists in this unity of sense.

The thalami optici have a yet more important office, and are associated in operations which rise still higher in the psychical scale. With Dr. Carpenter, I believe them to be the seat of

* *Vide* Dr. Laycock's paper "On the Reflex Function of the Brain," read at the meeting of the British Association, held at York, 1844.

those inner sensibilities and feelings which are associated with the emotional states.

Lying within the band of the corpora striata, the thalami, like these bodies, are in most intimate and extensive relationship with the cerebrum, through the instrumentality of innumerable fan-like commissural fibres,—Reil's nerves of the internal senses, —the *connecting links of thought with feeling*, and of *ideation* with *emotion*.

Along these channels, sensory impressions are transmitted upwards from the thalami to the perceptive organs, for *ideation* and *registration;* and from the cerebrum, ideas, thoughts, and the workings of ideo-dynamical, emotional, and mental agencies, pass downwards to them, there to receive these varying hues and shades of *feeling;* for, as Dr. Carpenter justly mentions, *thought* bears to *feeling*—the *cerebrum* to the *thalami*—the same relation which the physical impressions upon the organs of the external senses bear to the special endowments of their sensory ganglia in the encephalon; for instance, as in the sense of vision, the retina of the eye to the corpora quadrigemina.

I cannot dismiss the consideration of the thalami optici and corpora striata, *the great encephalic centres of sensibility and of motion*, without citing the authority of Dr. Todd. To Dr. Noble's hypothesis, that the corpora striata, with the optic thalami, form the special region of emotional sensibility, I do not subscribe:—

"The anatomy," says Dr. Todd, "of the corpora striata and optic thalami, while it denotes a very intimate union between them, also shows so manifest a difference in their structural characters, that it cannot be doubted that they *perform essentially different functions.* In the corpora striata the fibrous matter is arranged in distinct fascicles of different size, many, if not all of which, form a special connexion with its vesicular matter. In the optic thalami, on the other hand, the fibrous matter forms a very intricate interlacement, which is equally complicated at every part. Innumerable fibres pass from one to the other, and both are connected to the hemispheres by extensive radiations of fibrous matter. The corpora striata, however, are connected chiefly, if not solely, with the inferior fibrous layer of each crus cerebri; whilst the optic thalami are continuous with the superior part of each crus, which is situate above the locus niger. It will be observed, then, that while these bodies possess, as a principal character in common, their extensive connexion with the cerebral hemispheres, or, in other words, with the convoluted surface of the brain, they are, in the most marked way, connected inferiorly *with separate and distinct portions of the medulla oblongata;* the *corpora striata with the inferior planes of the crura cerebri and their continuations, the anterior pyramids;* and *the optic thalami with the olivary columns, the central, and probably fundamental portions of the medulla oblongata.* And this anatomical fact must be taken as an additional proof of their possessing *separate functions.*

"Now, it may be inferred, from their connexions with nerves chiefly of a sensitive kind, that the olivary columns and the optic thalami, which are continuous with them, are chiefly concerned in the reception of sensitive impressions, which may principally have reference merely to informing the mind (so to speak) or partly to the excitation of motion, as in deglutition, respiration, &c. The posterior horns of the gray matter of the cord, either by direct continuity with the olivary columns, or their union with them through commissural fibres, become part and parcel of a great centre of sensation, whether for mental or physical actions; and this leads us to view the thalami optici as the *principal foci of sensibility*, in intimate connexion with the convoluted surface of the brain, through its extensive fan-like radiations, and without which the mind could not perceive the physical change resulting from a sensitive impression. Again, the pyramidal bodies evidently connect the gray matter of the cord (its anterior horns?) with the corpora striata; and not only these, but also the intervening masses of vesicular matter, such as the locus niger, and the vesicular matter of the pons and of the olivary columns; and, supposing the corpora striata to be the centres of volition in connexion with the convoluted surface of the brain by their numerous radiations, all these several parts are linked together for the common purposes of volition, and constitute a great centre of voluntary actions, amenable to the influence of the will at every point."*

The fourth pair of vesicles in the human embryo are the Corpora Quadrigemina; but these are not simply the ganglia of vision (which function, as I have previously observed, some physiologists have restricted to the corpora geniculata); for, like the thalami optici, they have a higher and a wider range of action, and are manifestly the seat of those *objective emotional feelings* and *motor impulses,* which are roused into activity through the agency of sight. Of this fact we have daily and familiar illustration and proof in the infant's laughing eye, and in its expression of joyous emotion, as the perceptive consciousness begins to dawn; we see it in the effect produced by making strange faces at young children; we hear it in their scream of excited alarm, and we behold it in the convulsive fit, or shuddering agitation, which sometimes follows. Now, it is worthy of remark, that, in the brain of the fish, the corpora quadrigemina and the thalami optici are contained in one mass, forming the optic lobes; and this fusion, or binding up together of these ganglionic centres, is instructive, as indicating, at least, the closeness of the union, if it does not establish an identity of function. In the case of the young woman to whom I have already referred, in whom the intellectual faculties were in a state of abeyance, and whose only

* "Physiological Anatomy and Physiology of Man." By Dr. Todd and Mr. Bowman. Pp. 347, 348.

media of communication with the sensational consciousness were through *sight* and *tactile feeling, or touch,* we have in evidence, that through either of these channels, equally and alike, feelings of terror and of fright were *most* readily exerted; and assuredly this points to a *common centre* as the seat of these feelings, or to *an identity* and *unity* in the functions of the ganglionic centres concerned—the corpora quadrigemina and thalami optici. A more striking illustration, perhaps, cannot be found upon record, of the susceptibility to emotional excitement, than this young woman's case presents, at a time when the mental faculties were quite suspended.

And in relation to it, I may here reiterate what I have elsewhere stated, that

"While, on the one hand, it is abundantly manifest that the corpora quadrigemina are the seat of those *objective emotional feelings* and *motor impulses* which are roused into activity through the instrumentality of sight, I think, on the other, we may fairly, and are entitled to infer, that the thalami optici—the seat of our inner sensibilities—are the common centres of all our *other objective* and *subjective feelings*, and *motor impulses*, associated with the emotional states."*

For though it cannot be denied that simple emotional feelings and motor impulses may be, nay, easily and constantly are, excited and roused into activity, through all the special senses, by impressions from without, it must never be forgotten that the thalami optici are the common foci of sensibility for all the nerves of special sense,—the points of unity around which our different sensations are marshalled, and where they all centre and meet.

The Cerebellum is the last in the series of the encephalic ganglia. Placed over the divergent strands of the medulla oblongata, and consisting of a median lobe and two lateral appendages, it is in most intimate connexion with the apparatus of automatic life. In the adult brain no part of the encephalon has such extensive connexions with the cerebro-spinal axis, for it is in union with each segment of the great nervous centres upon which the sensations and movements of the body depend, but it has *no direct connexion with the cerebrum.*

The complexity of its structure induces the belief of a plurality of functions. The restiform columns derived from the posterior strands or columns of the spinal cord, there is every reason to infer, have the same endowments as the rest of the sensory tracts; and if the corpora dentata be the ganglionic centres in which they terminate, they must be centres of sensation closely allied to that of common or tactile sensation. *They are the seat of the muscular sense;* and, as Dr. Carpenter has suggested, the cere-

* "Physiological Psychology," page 36, *ante cit.*

bellum may only react (by reflex action) upon the impressions submitted to it, without being itself the instrument of communicating such impressions to the consciousness.

Comparative anatomy, pathological researches, and experimental inquiry, alike establish the position that the office of the lateral lobes of the cerebellum is the co-ordination of voluntary and locomotive actions; and whilst, on the one hand, the direct structural connexion which subsists between these co-ordinating organs—the lateral lobes and the corpora quadrigemina—clearly indicates the importance of the guiding influence of the visual sense in co-ordinated movements, so, on the other hand, analogous to this, is the influence of the restiform bodies, as channels for the transmission upwards to the corpora dentata, of impressions appertaining to the muscular sense.

The median lobe of the cerebellum is primitive and fundamental, exercising an independent function, since in the lower classes of the animal series up to birds, *the lateral lobes do not exist.* Pathological investigation has led me to espouse the opinion of Serres, that the median lobe is *the sensory ganglion of the sexual instinct.* Nor to this allocation of the generative propensity—which must be admitted to be one of the most universal instincts in nature, having for its object the perpetuation of the species—can I see a single valid *anatomical or physiological* objection, but on the contrary, from the intimate relations of the median lobe with the centres of sensation and emotional feeling, and through them with those of intellectual action, a clear and satisfactory explication of the complex character of the amative propensity in man.*

* I brought this view of the subject under the notice of the Royal Medical and Chirurgical Society, in a paper on "A Case of Apoplexy of the Cerebellum," read March 13, 1849, and published in vol. xxxii. of the Society's "Transactions." The case was that of a printer and publisher, fifty-two years of age, who died suddenly in an apoplectic attack, after having eaten a hearty dinner. At the autopsy there was found in the interior of the right hemisphere of the cerebellum an apoplectic clot, of the size of a pullet's egg, from a rupture of one of the branches of the vertebral artery. The whole arterial system of the brain was more or less in an unhealthy state, presenting that diseased condition of the vessels, which results from cartilaginous and ossific deposition between their coats. The interior of the hemisphere had become a softened pulpy mass, and the softening had extended inwardly beyond the centre of the median lobe, implicating the fibrous strands of the middle and inferior planes in the destructive process, and outwardly so near to the surface of the hemisphere, that a portion of the apoplectic clot was projecting through it.

Five months previous to the fatal seizure, he had a slight attack. I then found him low and exhausted, with a feeble pulse, and a cold clammy perspiration upon the surface of the body, complaining of sickness of stomach, and of pain, heat, and uneasiness in the back part of the head. He rallied in the course of the day, but the pain, heat, and uneasiness of the head continued for some days afterwards. There was no paralysis, but there was about him a hurriedness of manner, great restlessness, and irritability of temper. A few days afterwards, his wife told me, with great delicacy and embarrassment of manner, that he had become the subject

And it may truly be said that—

"An instinct of absolute necessity in its object is thus rendered a principle of our moral constitution, and connects itself with all our of a constant desire for sexual intercourse. His behaviour in this respect was so different from what it had been, and so little amenable to persuasion or reason, that she said necessity had induced her to speak on the subject to me. I at once recommended his removal from home, and succeeded in persuading him to pay a visit to some friends in the country, without his wife, on the plea that a change of air and scene was essentially necessary for the re-establishment of his health. He was absent about three weeks, and returned apparently improved in his general health, and no longer a slave to the sexual propensity. In this respect he had greatly changed. The desire for sexual intercourse had abated, and from that time it gradually became less and less up to the period of his death, while, at the same time, there was observed an unsteadiness in his gait, which visibly increased, and amounted at times, under the influence of emotional excitement, to the staggering of a drunken man; and, for some time before he died, he had a settled weakness and stiffness in the left leg and foot.—The condition of the cerebellum viewed in connexion with the history of the case, is full of interest. For while it is obvious that such an extensive disorganization of its internal structure must have destroyed the integrity of the functional powers of the part, it is highly interesting to note, during the progress of the degeneration, first, the exaltation and subsequent depression of the generative function, and, secondly, the tottering gait, from the defective power of co-ordination, ending in a weak and stiffened limb. As the extraordinary excitement of the sexual passion was a sudden invasion, and as this was manifested so soon after his first attack, the inference appears to be indisputable, that it was a consequence of that attack, and dependent upon the *co-relative stage* of that destructive disturbance of the cerebellum which led to such extensive degeneration of its structure. This is not the only case in which, from personal observation, I have been able to associate exaltation and subsequent depression of the sexual propensity with opposite pathological conditions of the cerebellum; the first with irritation and incipient inflammatory indications, and the latter with degeneration and abscess.

In one instance which came under my observation some years ago, the patient, though doatingly fond of his wife and children, and in every other relation of life an exemplary man, could not restrain the sexual passion; and I had him two or three times to treat him for gonorrhœa,—on one occasion during the period of his wife's accouchement. He wept over his delinquencies. In his case, at the latter period of his life, and after all erotic manifestations had passed away, he had not only a tottering, but a stooping gait, and required a walking-stick for support in progression. After death, there was found an extensive softening in the middle and in one of the lateral lobes of the cerebellum, as well as superficial ulceration of the glans penis, and atrophy of the genital organs. During the past year, an interesting case of tubercle of the brain in the adult came under my notice; and not the least remarkable of its features was the excited state of the generative function in the latter period of the patient's life. He died in a state of coma, from serous effusion at the base of the brain. In him the symptoms were so well marked, that during life tumour or tubercle on the brain was diagnosed. At the *post mortem* inspection, there was found, underneath the tentorium, quite unattached, excepting in its vascular connexions, a tubercular mass, about the size of a walnut, strikingly resembling the mulberry calculus, but having its base, which was about the size of a shilling, perfectly smooth; and corresponding to which was a depression, into which it was received, of some perceptible depth, on the surface of the left lobe of the cerebellum, very near to the median lobe. There was no lesion of structure, but all the surrounding parts were much congested. The pain was localized, and the paroxysmal attacks at times were very distressing. During life there was no paralysis, or loss of co-ordinating power. More than once he was urged to have a change of air and scene, but he would never stop away from home beyond a few days, as his wife could not accompany him—*he had no enjoyment at night without her.*

moral responsibilities, while, at the same time, it furnishes materials for those powers of imagination, taste, and perception of beauty, which, if not altogether peculiar to man, are at least his possession in degree infinitely above all that can be admitted into the comparison."*

After this general review of the nervous centres of the encephalon, we may now revert to the consideration of the crowning ganglia of the whole series—*the cerebral hemispheres,* the seat of the perceptive consciousness, of intellectual action, and volitional power,—in a word, of the understanding and the will. In the lowest of the vertebrate series of animals, the representatives of these hemispheres are limited to the anterior lobes, and reduced to mere lamina or crusts. But they gradually increase in size, complexity of structure, and in the number of their lobes and convolutions, as the animal rises in the scale of intelligence, until they reach their culminating predominancy in man. Professor Retzius, of Stockholm, has elaborately investigated the development of the cerebrum in the ascending vertebrata, and its different phases in the human embryo. His observations completely confirm the statements of Tiedemann and Serres as to the order in which the different lobes are evolved, showing that the *anterior lobe only* exists in fishes, that this enlarges as we ascend through the classes of reptiles and birds, but does not change its character; that the middle is not developed until we reach the mammalian class, presenting itself first in a very rudimentary form, and attaining increased development as we ascend; that the posterior lobe is developed from the back of the middle lobe, making its first appearance in the carnivorous group. To this history the embryonic development of the human cerebrum presents an exact parallel, the anterior lobe making considerable progress before the middle begins to be evolved, and the posterior being the latest in the order of succession.†

He gives the following account of the development of the cerebral hemispheres:—

"In the first period, which corresponds with the second or third months, only the anterior lobes form; in the second period, which is comprised in the end of the third, in the fourth, and in a small portion of the fifth, the two middle lobes appear, and after this time the posterior lobes. During a great portion of the first period, the descending horns of the lateral ventricles and the pedes hippocampi are wanting: these are added in the second period. During a great portion of the

* Sir Henry Holland's "Chapters on Mental Philosophy," page 216.

† I would here take the liberty of suggesting to others, who are engaged in the practice of midwifery, and who feel interested in psychological inquiries, that they should allow no opportunity to escape them of inspecting the state and observing the phases of the embryonic development of the brain in cases of abortion, and thus of verifying, as I have had repeated opportunities of doing, some of the concurring statements of Tiedemann and Retzius, by the test of their own personal observation.

first period, the hemispheres do not cover the thalami optici; in the second period they completely overlap these parts, approach the large corpora quadrigemina, cover their anterior part, and then descend by the side of the cerebral nucleus (cone and stem), and, as it were, fold round it. If we examine a brain at this period of development, we might, from its external appearance, imagine that the posterior margin of the hemispheres corresponds to their persistent posterior ends and margin, that is, to those which are their posterior margins in their perfectly developed state. But it is not so. If we open the brain we come at once to the descending horns of the lateral ventricles, in which are the rudiments of the great pedes hippocampi. At a later period, in the fourth month, a small superficial notch is formed at the posterior margins of the hemispheres; and that part of the margin which is above the notch, is the first rudiment of the posterior lobes of the hemispheres. These, which are thus for a time only rudimental, begin above the middle lobes, gradually take in their posterior margin, follow it down, as development advances, by the sides of the cerebral nucleus, and terminate in that part of the *middle lobes* which meet the pedes hippocampi. Even in the brain of the mature fœtus, as well as in the fully developed brain of older persons, the posterior lobes are very clearly separated from the middle lobes by a branching furrow, which is especially distinct on the vertical side of the hemisphere which lies next to the *falx*."*

This tripartite division of the cerebrum into distinct lobes, and the order and succession of their development, are points of great psychological significance; for the observed facts clearly indicate that the cerebral lobes are evolved from *before backwards*, in the order and degree of their importance as psychical instruments, and they point to the middle and posterior lobes, but especially to the latter of these, with peculiar interest. It is only in man that we meet with such a great development *backwards* of the posterior lobes, and that the cerebellum is completely overlapped and covered by them. The anterior lobes are remarkable for their great extension *forwards;* but it must be conceded that the chief distinction between the cerebrum of man and that of the higher mammalia, is much more striking in reference to the *posterior* than to the *anterior* lobes. "The brain of the chimpanzee," says Professor Owen, "in the relative proportions of the different parts, and the disposition of the convolutions, especially those of the posterior lobes, approaches nearest to the human brain: it differs chiefly in the *flatness* of the hemispheres, in the *comparative shortness of the posterior*, and in the *narrowness* of the anterior lobes."

I am fully aware that some physiologists maintain that this tripartite division of the cerebrum into lobes is altogether arbitrary and useless; and it cannot be denied that it is quite im-

* Forbes's British and Foreign Quarterly Medical Review, vol. xxii. p. 503.

possible, when we survey the cerebrum from above, to point out where the second lobe ends and the third begins; for there is no breach in the continuity of the surface, but between the first and second the *fissura Sylvii* presents a line of demarcation sufficiently distinctive, and on turning the base of the brain upward we at once see the meaning of these divisions.

No one, however, can make any such survey of the brain, without being struck with the appearance and character of its convolutions.

A classification of these, begun by Professor Owen, has been greatly extended by M. Leuret; and it is much to be regretted that he did not live to complete his elaborate and valuable researches. The subject is one of great interest and vast importance; for it is an indisputable fact, that the complexity of these convolutions is an index to the place which the animal holds in the scale of intelligence. "Observation," says Leuret, "has shown what strict induction had led us to conclude, that each group of brains among animals has a type proper to it, and that the type is characteristically manifested by the form of its convolutions." Every family has a brain formed in a determinate manner; and the number, form, arrangement, and relations of the convolutions are found to be in strict accordance with the intelligence displayed. He justly makes a distinction between those convolutions which are *primary* and *fundamental*, and to be found throughout the whole series of convoluted brains, occupying the same position, and differing only in their size and extent,—and those *secondary* convolutions which are not constant, even in brains of the same group of animals, but are dependent upon the extent of the primary ones, and the connections which they form with others that are near them.*

* Gall was the *first* who *classified* the *convolutions;* and the labours of Gall, Spurzheim, and Holm in this interesting field of inquiry were great and manifold: and I would here take the opportunity of paying a passing tribute of respect to the memory of Mr. H. H. Holm, the friend and pupil of Spurzheim, who studied comparative cerebral anatomy with great enthusiasm. He was a Fellow of the Zoological Society, and, residing near the Society's menageries, he had easy access to the collection, of which he availed himself, to study the habits and dispositions of the animals; and having permission to examine the crania and brains of those which died, his anatomical and physiological researches were rightly carried on.

Professor Owen, in his valuable paper "On the Anatomy of the Chetah," (*Felis Jubata,*) communicated to the Zoological Society on Sept. 10, 1833, and published in the first volume of the Society's Transactions, gives a note from Mr. Holm, containing his opinions of the *functions* of the different convolutions in the brain of the chetah, on a comparison of it with the human brain and that of some other animals. After an elaborate description of the brain of the chetah, Professor Owen says—"Of the constancy of the disposition of the convolutions represented by Gall and Spurzheim as characteristic of the brain of the feline genus, I was first assured by our fellow-member, H. H. Holm, Esq., Lecturer on Phrenology, whose attention has long been directed to this part of anatomy." Mr. Holm was a Member of the Royal College of Surgeons, but, enjoying an independence, he

To determine the functions of the primitive convolutions *is the great problem of physiological psychology,* and as I have elsewhere* observed, *it remains unsolved.* Nor is this surprising when we consider the conditions of the problem. We are required carefully to note the first appearance and progressive development of the primitive and fundamental convolutions from below upwards in the ascending series of animals, and to endeavour to analyse with certainty the characters of different animals, in relation to the objects of their intellectual faculties, in accordance with the cerebral convolutions as contrasted with mere consensual actions. Like things are to be compared with like, convolution with convolution, and the same groups in different animals with each other, before the problem can be solved.

All honour is due to Gall, for he was the first to enunciate clearly the true relations between the psychological nature of man and that of the lower animals; and while we claim for Unzer and Prochaska the defining of the boundaries of the *sensorium commune,* we must look upon Gall as the founder of physiological psychology. One of the most remarkable men of the age in which he lived, he was alike distinguished for originality and independence of thought, for powers of observation, untiring industry, and indomitable perseverance. To him and his able coadjutor, Spurzheim, medical science, as well as physiology and psychology, is under great obligations. And it is no detraction from their merits to reconsider, if not to remodel, the system of organology which they propounded, by the light which subsequent physiological inquiry and discovery have thrown upon the subject. In the prosecution of such inquiries, the inductive philosophy of Bacon must be our guide. For while it is never to be forgotten that a refined analysis discovered the harmony of the celestial motions, and conducted the immortal Newton through a maze of intricate phenomena to the great laws appointed for the government of the universe, it is melancholy to reflect for how many ages the opinions of one man were the measure of truth and reason, and, under the sovereignty of the sway of the Stagirite, how universal was the degradation of the human understanding.

devoted himself to the pursuit of phrenology, instead of entering upon medical practice. His lectures were amply illustrated by casts, crania, and brains. He pointed out the cerebral convolutions which constitute the several organs, described the modifications which the convolutions receive, and compared them together to illustrate their magnitudes, positions, junctions, and outer connections with great ability; and so highly did Dr. Spurzheim estimate his talents, knowledge, and zeal, that he made him the special depositary of his latest views on the configuration of the cerebral organs in man and the mammalia. Unfortunately, like Leuret, he was cut off in the midst of his labours, and in the fortieth year of his age.—*Vide* a Biographical Notice of Mr. Holm in vol. xix. "Phrenological Journal."

* "Physiological Psychology," page 48.

But still it is gratifying after the lapse of ages to behold the father of experimental philosophy, the illustrious Bacon, clearly pointing out the absurdity of pretending to account for the phenomena of nature by syllogistic reasoning on hypothetical principles, and with a boldness becoming a genius of the first order, undertaking to give a new chart of human knowledge. Let us follow its guidance and tread in his footsteps. Already there are many labourers in the field, and much has been accomplished. A second Newton may arise among them to thread the labyrinth of metaphysical subtlety and transcendental philosophy with the logical acumen of a Locke, to collect and bind together the scattered and isolated links of the great chain of physiological discovery, to point out the bearings of the pathological facts of past experience, to interrogate nature herself upon the functional characters "written upon the nervous pulp" of the several ganglia, and to read her own replies in the living experiments which she has presented to us in the lower forms of animal existence, and thus to place the great doctrines of mind on the solid basis of a sound physiological psychology!

Since the enunciation of Gall, that the convolutions of the cerebrum are the seat of the faculties of the mind, their development and classification has been invested with peculiar interest.

"Anatomy," says Dr. Todd, "points to the conclusion that the office of the convolutions is connected with the functions of the mind; and it seems not improbable that the phrenological view which assigns to *certain convolutions* a special office connected with some particular faculty or faculties is true. This is strongly supported by the fact of a regular disposition of certain *primary* convolutions, and that, in tracing the convolutions from the most simple to the most complex, indications are found of the *persistence* of the *primary* and *fundamental* convolutions in the midst of many that are secondary and superadded ones."*

M. Leuret has shown, that in all the inferior classes of animals up to the lowest mammalia, the cerebrum is not convoluted on the surface. In the bat, the mole, and the rat, &c., as in birds, the cerebral hemispheres are perfectly plain and smooth, though divided by the Sylvian fissure; and among the earliest to appear, are the convolutions of the insula of Reil, in the fissura Sylvii. In the rabbit, beaver, and porcupine, the Sylvian fissure is strongly marked, but there are only a few slight depressions indicating the future sulci of the convolutions on the surface of the hemispheres. In the fox, wolf, and dog, the simplest form of the true convolutions are first met with,—*the fundamental convolutions of Leuret.* In the fox, as a typical example, they are six in number. Four of these are on the external surface, running

* Dr. Todd's "Cyclopædia of Anatomy and Physiology."

from *before backwards*; one forms the curved lip, or border of the Sylvian fissure, and surrounds the island of Reil; the other three, also carried in this direction, are placed parallel to the first, and one above another; the fourth or superior longitudinal, occupies the margin of the great longitudinal fissure; the fifth, situated anteriorly, under the forepart of the anterior lobe, is the super-orbital convolution; and the sixth is the great internal convolution, above the corpus callosum—la circonvolution de l'ourlet of Foville.

In the human brain, besides the great internal and the super-orbital convolutions, M. Leuret has represented *three external fundamental convolutions*, which are tortuous, and frequently communicate with each other. Between the anterior and posterior portions of these three external convolutions, are interposed, on the upper surface of the hemispheres, two sets of transverse convolutions, divided by a distinct sulcus, which runs outwards and forwards, from the longitudinal fissure, so that the right and left grooves form a V-shaped line, open in front, which is called by Leuret, the fissure of Rolando.

These transverse superior convolutions are peculiarly characteristic of the human brain; and to this peculiarity must be added the elongation *backwards* of the cerebrum, by the increased development of the posterior lobe, and the greater and marked complexity of the vertical convolutions in the median fissure, and of those of the island of Reil.

In the development of the cerebrum, Foville has invested the *locus perforatus anterior*, or quadrilateral spot, with paramount importance, as being the central nucleus and fundamental part of the brain,—the starting point from whence the primitive convolutions are evolved; and thus makes it the portal to intellectual action and volitional power.

Of the primitive convolutions he makes four orders, in each of the cerebral hemispheres, distinguishable one from another. Of these, the first order contains but one—the convolution of the band,—the ourlet or hem of the hemispheres,—the great internal convolution. It surrounds the hemispheres internally like a riband, and is attached at each extremity to the *locus perforatus*. *It is clearly the basement convolution of the cerebrum.*

The second order are the marginal convolutions, of which there are two. One, the great longitudinal convolution, occupies the circumference of the hemisphere, forming its excentric or outer boundary, while the other surrounds the insula of Reil and the *fissura Sylvii*. They arise from the quadrilateral space, and from the convolution of the band, from which they spring like buds from a branch.

The great marginal convolution of the longitudinal fissure forms the inner border of the triangular orbital surface of the anterior lobe, where cleft, as it were, in twain, it receives in a deep sulcus the olfactory nerve; the outer border of the triangular surface is formed by the marginal convolution of the fissura Sylvii, and at the apex of the triangle behind, the two borders are connected by a short and but slightly elevated convolution, bounding the locus perforatus anterior in *front*.

The convolutions of the third order, of which there are two sets, are situated on the *internal* surface of the hemispheres, forming a sort of anastomosis between the convolutions of the first and second order. These hook-like processes on the convolution of the band, led Rolando to call it *processo cristato*. The second set are within the fissura Sylvii, and constitute the insula of Reil.

The convolutions of the fourth order, the largest, deepest, and least symmetrical of all, are quite detached from the perforated spot, and have no *direct* connexion with the convolutions of the first order. They occupy, in a transverse direction, the outer or convex surface of the hemispheres, and they thus connect the two convolutions of the second order together—viz., the marginal convolution of the median fissure and that of the fissura Sylvii. They are especially characteristic of the human brain, and may be considered "as prolongations of the convolutions of the third order, below the two convolutions of the second order, and running directly across the upper surface of the brain."*

We may now proceed to the determination, if we can, of the organ of the perceptive consciousness in the cerebrum, and then resume the consideration of the phenomena which formularize the perceptive consciousness—namely, ideation and volition, with their associates, memory and emotional sensibility. Perception is the correlative of sensation, and indicates its intellectual phase; for, in this second stage of our psychological development, we have *intelligent ideas*, *emotional feelings*, and *volitional actions*.

I quite agree with Dr. Todd, that the psychologist must determine what are and what are not fundamental faculties of the mind, before the physiologist can venture to assign to each its local habitation in the brain. But about the perceptive consciousness there can be no dispute, and to my mind, quite as little about the existence of a central organ in the cerebrum, as its local habitation and instrument—the seat of ideation and volitional powers. In the nervous apparatus of the sensational consciousness, we have seen that there is a central organ—a point of unity around which the various sensations are marshalled, and that the thalami optici are these central foci of sensibility,

* Solly "On the Brain," page 136.

"without which the mind could not perceive the physical changes resulting from sensitive impressions." So, too, the perceptive consciousness has its central organ, where ideation is effected, whence issue the mandates of the will, and where sensory impressions—the intuitions of the special senses—are translated into the form of intelligence, and become intellectual phenomena —are perceived and associated, and where the intuitions of one sense are used to correct and elucidate those of another. But the question recurs, Can we determine the site of the organ of the perceptive consciousness? Do embryology and comparative anatomy afford us any clue to the solution of the question, or throw any light upon the subject? I think they do. For if we revert to the phases of embryonic development, we find, about the tenth week, that the central nuclei of the cerebral hemispheres, from being at first mere points, then actually cover the corpora striata, after the manner in which they permanently incrust those bodies in the brain of the full-grown fish, so that we cannot avoid the conclusion, but are legitimately led to infer that the *latter* are the homologues of the *former*. Now, if this be conceded, the induction is irresistible as to the site of the organ of the perceptive consciousness in the cerebrum. For, wherever the hemispherical ganglia exist, the essential phenomena of the perceptive consciousness are manifested; and since it is admitted, and without a moment's hesitation, by every experienced angler, that in the case of the fish we have clear and distinct evidence of the exercise of the perceptive faculty of memory and volition, *as opposed to mere consensual and instinctive action,* there can be no dispute that the thin laminæ of vesicular matter which incrust the corpora striata in the brain of the fish, are the organs of its perceptive consciousness. But in the human embryo it is equally clear and indisputable that these thin laminæ of vesicular matter are the primitive and basement convolutions of the hemispheres —the convolutions of the band—the ourlet of Foville, and ultimately *the great internal convolution* of the adult brain; so that if the former be really the homologues of the latter, the inference is most important and indisputable as to the seat of the perceptive consciousness in man. Moreover, one thing is abundantly manifest, that since these great internal convolutions are unquestionably the primitive basement convolutions of the hemispherical ganglia, they must be the *portals* to *intellectual action*, where sensory impressions are translated into the form of intelligence, are perceived and idealized. It was here I believe that Gall located his organs of individuality; but since these convolutions are manifestly the portals to intellectual action, and as perception is but one and the first step above sensation, I think we are fully warranted in taking a more comprehensive view of their formation, and in considering them the organs of the perceptive consciousness, —the seat of *ideation*, *memory*, and *volition*. Now, of all the convolutions of the brain, they are the most symmetrical; they are the most constant and regular, and each exhibits with its fellow on the opposite side the most exact symmetry. Their connexions are multitudinous, and commensurate with their importance. Besides their relations with the sensory ganglia of special sensation, first and anteriorly they are in intimate connexion with those super-orbital convolutions of the anterior lobes, to which pathological investigations point as the organs through which we acquire a knowledge of the physical adjuncts of external existences, such as their size, shape, colour, number, weight, or resistance, &c.: secondly and laterally, they are connected with those primitive and early developed basilar convolutions surrounding the fissura Sylvii, which, from their connexion with the earliest of the animal senses, that of smell, appear to administer to the universal instinct of self-preservation: thirdly and posteriorly, they are in intimate union with those backwardly developed convolutions of the posterior lobes which belong more exclusively to the family of man: fourthly and superiorly, they are connected, through an order of anastomosing convolutions, with those great marginal convolutions which constitute the outer and most exalted boundaries of the hemispheres, and with those which take a longitudinal but tortuous course on the upper and outer surface of the brain, thus connecting, as it were, perception, the first step above sensation, with the loftier regions of thought.*

Now it is only in the human brain, that these basement convolutions of the hemispherical ganglia exist in the highest state of development. Compared with what we meet with in the brain of the monkey and other anthropomorphous animals, the

* "Of the internal convolution, or that of the corpus callosum, called by Foville, *convolution d'ourlet* (*processo cristato*—Rolando), the principal portion is above and parallel to the corpus callosum: in front it curves down parallel to the anterior reflector of the corpus callosum, as far as the locus perforatus, connecting it with some of the *anterior convolutions*. Behind it passes in a similar manner round the posterior reflection, connecting itself with some of the *posterior convolutions*, and in the *middle lobe* forming the hippocampus major, the anterior extremity of which is situate immediately behind the fissura Sylvii and locus perforatus. Its horizontal portion appears to be connected with some nearly *vertical* ones, which seem indeed to branch off from it. It forms, to use Foville's expression, a *hem* or *selvage* to the cortical layer of the cerebral hemispheres. The free margin of this convolution varies its character in different brains, according to the degree of tortuosity it exhibits, and the number of small fissures which are met with in it. The small folds which connect it with other convolutions on the inner surface of the hemisphere vary in number, and are generally found most numerous in the posterior part. Some of these folds are not distinctly visible unless the sulcus above it has been freely opened, as they are situated quite on its floor."—(Dr. Todd, on the Physiology of the Nervous System, "Cyclopædia of Anatomy and Physiology," p. 697.)

contrast is not more striking than it is psychologically significant. In man these complications and relations with the other primitive convolutions of the cerebrum are commensurate with their importance and with the vast and varied range of their function as psychical instruments. For while in the perception proper of outward existences, man stands on the same platform with the lower animals, and the process in him, as in them, is equally and alike intuitive, nature is not to him a mere system of *shapes, shades*, and *resistances;* but, by virtue of his highly attuned organization, "it speaks to him a higher language, embodies loftier ideas, and breathes into the soul diviner sentiments."

Perception has been aptly designated world-consciousness. For while in sensation the conscious mind is solely absorbed in its own subjective conditions or feelings, as induced by the bodily states, in perception its attention is transferred from *these* to their *interpretation*, as *expressive* of outwardly existing facts, and thus it implies a consciousness of the object which induced the sensation or impression,—a recognition of its cause, as a something external to the mind itself,—*an outward reality.* So that while sensation, on the one hand, is wholly subjective, in relation to knowledge, perception is, on the other, objective. In other words, the one is *self*, and the other is *world-consciousness.*

"But self-consciousness and world-consciousness are indissolubly connected. The one cannot exist without, but only by the other. Self is first perceived as that which is not *phenomenon;* the world is first perceived as that which is *not self.*"*

But self-consciousness is the primary condition; for, as we have already seen,—

"The mind at first exists simply upon its sensational stage of development, and it only gradually, through the various impulses exerted by all the variety of subjective impressions, struggles out of *self*, and sees both *self* and *nature* in clear opposition. At first, however, it cannot interpret all these impressions in relation to its newly-acquired *world-consciousness.* This is the work of time and experience. Trace after trace has to be laid up in the mind, many of them to be compared together; the intuitions of one sense to be used in correction or elucidation of another; and thus gradually the *sign language* of *sensation* has to attain to the meaning which we attach to perception."†

All our perceptive experience is thus idealized from fragmentary impressions made upon the sensory organs,—the perceptive faculty idealizing the impression and converting it into an intellectual phenomenon, or knowledge. For no sooner has the perceptive consciousness been awakened, than a sight or sound which before produced an involuntary start, now excites a

* Morell's "Psychology." † Ibid.

smile of recognition, the mind struggles out of self, beginning to throw itself into the objects around it, and to live in the world of outward realities. Mr. Morell has well observed,—

"Man is, at first, a mere creature of sensation and instinct; from that he rises to the power of perception, separating the world from himself, and becoming conscious, *here* of his own identity, *there* of the universe around him. After this, he attains to the power of representation and expression, stamps upon objects their distinctive names, classifies and generalizes them, and penetrates them with the light of the *understanding*. After this process of analysis, begins the still higher process of synthesis. The objects separated and classified, are now reconstructed in scientific order, and the truths which were first seen only by the light of sense and intuition, are now comprehended by the clearer light of *reason*. With the development of the *reason* are given the conditions for the development of the *will*, which rises, through like gradations, from mere instinct to conscious *self-action*, and, at last, to the height of *perfect freedom*."*

(*To be continued.*)

---

### Art. VII.—WILLIAM PALMER.

* Morell's "Elements of Psychology," page 59.

